# Multiple Mating in the Citrophilous Mealybug *Pseudococcus* *calceolariae*: Implications for Mating Disruption

**DOI:** 10.3390/insects10090285

**Published:** 2019-09-05

**Authors:** Renato Ricciardi, Andrea Lucchi, Giovanni Benelli, David Maxwell Suckling

**Affiliations:** 1 Department of Agriculture, Food and Environment, University of Pisa, via del Borghetto 80, 56124 Pisa, Italy; 2 The New Zealand Institute for Plant & Food Research Limited, PB 4704, Christchurch 8140, New Zealand; 3 School of Biological Sciences, University of Auckland, Tamaki Campus, PB 92019, Auckland 1142, New Zealand

**Keywords:** sex pheromone, biological control, flight tunnel, Integrated Pest Management, mealybug monitoring

## Abstract

The citrophilous mealybug *Pseudococcus calceolariae* (Maskell) (Hemiptera, Pseudococcidae) is a primary pest of various crops, including grapevines. The use of insecticides against this species is difficult in most cases because its life cycle includes an extended duration of eggs, juveniles, and adults under the bark and on the roots. Pheromone-based control strategies can present new eco-friendly opportunities to manage this species, as in the case of *Planococcus ficus* (Signoret) and *Planococcus citri* (Risso). With this aim it is critical to understand behavioral aspects that may influence pheromone-based control strategies. Herein, the capability of males to fertilize multiple females was investigated, trying to understand whether this behavior could negatively impact the efficacy of mass trapping, mating disruption, or the lure and kill technique. Results showed that a *P. calceolariae* male can successfully mate and fertilize up to 13 females. The copulation time in subsequent mating events and the time between copulations did not change over time but the number of matings per day significantly decreased. In a further experiment, we investigated the mate location strategy of *P. calceolariae* males, testing the attractiveness of different loadings of sex pheromone on males in a flight tunnel. Males constantly exposed to 16 rubber septa loaded with the sex pheromone showed a significant decrease in female detection at 1 and 30 μg loadings (0.18 and 0.74 visits per female for each visit per septum, respectively), whereas in the control about 9.2-fold more of the released males successfully detected the female in the center of the array of 16 septa without pheromone. Male location of females in the control (45%) was significantly higher than in the arrays with surrounding pheromone (5% and 20% at 1 and 30 μg loadings, respectively). Mating only occurred in the control arrays (45%). This study represents a useful first step to developing pheromone-based strategies for the control of citrophilous mealybugs.

## 1. Introduction

Vineyards host a great variety of pests worldwide which impact grapevine health in different ways [1]. Many of these pests belong to the order Hemiptera and especially to the family Pseudococcidae, commonly known as mealybugs [2]. They compromise grape quality by contaminating bunches with honeydew, which leads to the development of sooty mold fungi. Furthermore, they can transmit several important grapevine viral diseases [3]. Farmers usually rely on insecticide applications to manage these pests. However, mealybugs can develop resistance to currently marketed insecticides and new reports are still emerging [4]. Several species of mealybugs have become resistant to insecticides, which is unsustainable as this negatively affects their control [5,6,7,8,9,10,11]. However, to boost grape production under an environmentally-safe agricultural system, it is necessary to find alternative solutions to effectively manage crop pests while reducing insecticide overuse [12,13,14]. Hence, it is critical to develop more sustainable Integrated Pest Management (IPM) systems [15,16]. An alternative is presented in synthetic sex pheromones, which disrupt the chemical communication between male and female of a given insect species, thereby preventing mate location and mating [17]. This approach is currently known as sex pheromone-based mating disruption (MD). Each mealybug species relies on unique pheromone(s) for sexual communication, with structures characterized by an irregular non-head-to-tail monoterpenoid structure [18]. To date, the pheromones of 19 mealybugs species belonging to seven genera have been identified [19]. This approach can be very effective. For example, mating disruption of *Planococcus ficus* (Signoret) (Hemiptera, Pseudococcidae) usually leads to a significant reduction of the abundance of ovipositing females, an increase of the pre-oviposition period, lower damage rates on grape bunches, and thus an overall decrease of the pest population densities [20,21,22].

The citrophilous mealybug, *Pseudococcus calceolariae* (Maskell) (Hemiptera, Pseudococcidae) impacts upon grape production through the abundant emission of honeydew, with subsequent development of sooty mold and transmission of two closteroviruses, GLRaV-1 and GLRaV-3 [23]. Moreover, *P. calceolariae* is a cosmopolitan and polyphagous pest on different crops (orange, lemon, and avocado, as well as other vegetable, fruit, and floricultural plants) [24,25,26].

Notably, comprehensive studies on the basic biology of *P. calceolariae* have been carried out, also shedding light on its chemoecology. Rotundo and Tremblay [27] and Rotundo et al. [28] focused on the male flight activity, providing evidence of the release of sex pheromones. Later, Silva et al. [29] studied its mating behavior, showing that this mealybug is obliged to mate for reproduction [30]. The sex pheromone of *P. calceolariae* was identified [25], allowing for the synthesis of a synthetic pheromone. No evidence of habituation was detected in *P. calceolariae *males exposed to the sex pheromone (1 mg for 24 h); indeed, they were able to promptly locate the sex pheromone source despite earlier pre-exposure to the chemical [31]. However, even though Silva et al. [32] studied multiple mating in this mealybug species, little is currently known about the potential effect of this factor on monitoring and control.

Multiple mating occurs in many orders of insects, including moths such as *Epiphyas postvittana* (Walker) [33] and *Cydia pomonella* (Linnaeus) (Lepidoptera, Tortricidae) [34], as well in other mealybug species such as *P. ficus*, *Pseudococcus longispinus* (Targioni Tozzetti) and *Pseudococcus viburni* (Signoret) (Hemiptera, Pseudococcidae) [35]. Knowledge about the presence and frequency of multiple mating in a given pest species is timely and important. Indeed, a key issue to watch for is that the males are able to mate with several females, thus reducing the efficacy of several control tools, including mating disruption, mass trapping, and the lure and kill technique, which all have the goal of preventing mating or reducing it by removing as many males from the population as possible [26].

In this scenario, the present study provides insights into the reproductive biology of *P. calceolariae* by having investigated how many females can be fertilized by a single male within a day as well as during the whole lifespan. The effect of subsequent mating events on copulation duration and time elapsed between copulations has also been evaluated. By simulating mating disruption in the laboratory, we tried to assess the potential effectiveness of this technique to manage *P. calceolariae*, following recent successful attempts on another mealybug species (*P. ficus*) [14,21,22,36,37].

## 2. Materials and Methods

### 2.1. Insect Rearing

*Pseudococcus calceolariae* mealybugs tested here were obtained from a colony held at The New Zealand Institute for Plant & Food Research Ltd. (Lincoln, New Zealand). The new colony was maintained inside ventilated plastic containers (30 × 25 × 10 cm) and periodically fed with new potato sprouts (*Solanum tuberosum* L.). We did not use individuals from the field to avoid contamination of other species. Crawlers were collected every 3–4 days and separated in different boxes (10 × 7 × 7 cm) to obtain mealybug cohorts of different ages. After 15–20 days, mealybugs were sexed by separating male cocoons before they hatched to avoid possible mating (males were easily identified by the presence of a cocoon). Immature individuals were reared on fresh potato sprouts in separated ventilated plastic containers (10 × 7 × 7 cm). Each box contained 20–40 virgin females. We tested 24–32 h-old males; their sexual maturation was indicated by complete growth of the wax tail, as detailed also for *Planococcus citri* (Risso) [38,39].

### 2.2. Multiple Mating Experiment

To estimate the number of females that could be fertilized by a single virgin male, we followed the method proposed by Waterworth et al. [35] with minor modifications. Ten *P. calceolariae* females and a virgin male were kept close to each other for 6 h in a sterile Petri dish (35 × 10 mm). All replicates were recorded using a high definition webcam (HD C525, Logitech, Lausanne, Switzerland) placed above the arena and connected to a computer (OptiPlex 745, DELL, Round Rock, TX, U.S.). Recordings were analyzed to measure (i) the copulation duration (s), (ii) the copulation number per day, and (iii) the time interval between matings. Then, each female was moved to a Petri dish (35 × 10 mm) and provided ad libitum with potato sprouts. Oviposition activity was monitored daily to measure the time required to produce an ovisac, following Silva et al. [29]. The following day, surviving males were introduced into a new arena with a new group of virgin females; this procedure was replicated with 30 males.

All tests were conducted in a room (22.5 ± 1 °C, 40 ± 2.5% RH; 16:8 (L:D) photoperiod) illuminated with daylight fluorescent tubes to obtain a light intensity in the proximity of the arena of 1000 lux. Each trial was carried out between 8:30 and 14:30.

### 2.3. Flight Tunnel Experiments

Controlled tests were conducted in a laminar airflow flight tunnel as described by El-Sayed et al. [40]. For each trial, two *P. calceolariae* males were set in a plastic vial (20 mL). Before the start of the experiment, the vial was placed in the center of the floor of the flight tunnel and males were allowed to acclimatize to the flight tunnel conditions (22.5 ± 1 °C, 40 ± 2.5% R.H, wind speed 0.4 ± 0.1 m/s) for 2 min. Each trial lasted 60 min. The different loadings of pheromone used were chosen based on pre-exposure tests reported by Suckling et al. [31]. Three experiments were carried out, as detailed below.

Firstly, we measured the flight tunnel response of *P. calceolariae* males to increasing loadings of sex pheromone in a 4 × 4 grid composed by 16 lures baited with synthetic sex pheromone; in this way, we replicated, on a reduced scale, what happens in the field when MD is attempted. We determined whether the male ability to locate a female could be disrupted using an amount of sex pheromone that was much higher than that naturally released by the females in the field, having observed much higher trap catches to synthetic lures when compared with mealybug females [25]. This information, considering the knowledge acquired on the extent of multiple mating, is critical to fully understand the potential and effectiveness of pheromone-based control strategies, with special reference to MD.

#### 2.3.1. Trial 1: Male Response to a Single Loading of Sex Pheromone

To simulate the short distance between males and females in a field colony, we released two males close to a sex pheromone-loaded array of lures. The goal was to see which and how many lures were visited and whether they preferred the upwind or downwind lures. Herein, a 4 × 4 array of 16 rubber lures (Ø = 0.5 mm, h = 0.2 mm, 2 cm apart) was placed in the flight tunnel, with each lure loaded with 1 μg of synthetic pheromone; the pheromone was synthesized by the New Zealand Institute for Plant & Food Research laboratories according to the method described by El-Sayed et al. [25]. An A4 white sheet with a grid of 16 unloaded lures was used as a control. Herein, we tested both downwind and upwind directions (using different males every time) to evaluate the mealybug preference in terms of number of visits per lure. After confirming that the preferred direction was upwind, we compared all treatments upwind. With each new couple of males released 10–15 cm from the grid, the grid was rotated clockwise 90° to avoid positional effects [41]. We tested 30 male pairs.

#### 2.3.2. Trial 2: Male Response to Four Different Loadings of Sex Pheromone

In this experiment, we used the same grid used in trial 1; however, it was simultaneously baited with four increasing sex pheromone loadings from downwind to upwind. The aim was to understand whether males preferred certain loadings over others. As in the previous experiment, *P. calceolariae* males were released close to the grid. An A4 white sheet with 16 rubber septa (4 lines × 4 lines as above) was used with four different loadings of synthetic sex pheromone for each row: 1, 3, 10, and 30 μg, respectively, increasing upwind. A control with 16 unloaded lures was used. The odor sources were renewed after every 10 males tested. During each replicate, we observed which and how many lures were visited by *P. calceolariae* males. A total of 30 replicates were carried out.

#### 2.3.3. Trial 3: Mimicking Mating Disruption

Trial 3 followed the array setup of trial 1; attraction of males to a central virgin female surrounded with a control or either of two loadings of synthetic sex pheromone (each septum was loaded with 0, 1, or 30 μg of sex pheromone) was tested. By placing one virgin female in the middle of the array, it was possible to observe whether the lures were able to mask the presence of the female. The female was held with a small piece of double-sided tape.

During each replicate, we recorded: (i) which septa were visited by *P. calceolariae* males, (ii) the number of male visits per septum, (iii) the duration of each visit, and (iv) the mate location success of males attempting to locate a female in each treatment and control.

### 2.4. Statistical Analysis

In the multiple mating experiment, data about copulation duration, time between copulations and number of copulations per day were analyzed by JMP 11 [42] with a general linear mixed model (GLMM) with one fixed factor, i.e., the day of the observation [43]: y_i,w_ = μ + D_i_ + ID_w_ + e_iw_, where y_i,w_ is the observation, μ is the overall mean, D_i_ is the i-th fixed effect of the day of the observation (i = 1–3), ID_w_ is the w-th random effect of the individual over repeated testing phases (_w _= 1–30), and e_iw_ is the residual error. Means of treatments were separated by the Tukey’s HSD test. A *p*-value of 0.05 was selected as the threshold to assess significant differences.

In flight tunnel trial 1, the possible presence of significant differences among male visits to different rows of pheromone-baited lures was studied using a contingency analysis, highlighting that males randomly visited pheromone-baited lures in the flight tunnel. Therefore, data testing different pheromone loadings in the flight tunnel experiments (i.e., male visits per lure (n), female location events (n), and time spent on each lure (s)) were not normally distributed and it was not possible to normalize the distribution to homogenize the variance (Shapiro-Wilk test, goodness of fit *p* < 0.001). Therefore, all data were analyzed by Kruskal-Wallis test followed by Steel-Dwass test to make nonparametric comparisons between all pairs. A *p*-value of 0.05 was selected as the threshold to assess significant differences.

## 3. Results

### 3.1. Multiple Mating Experiment

**Figure 1:**
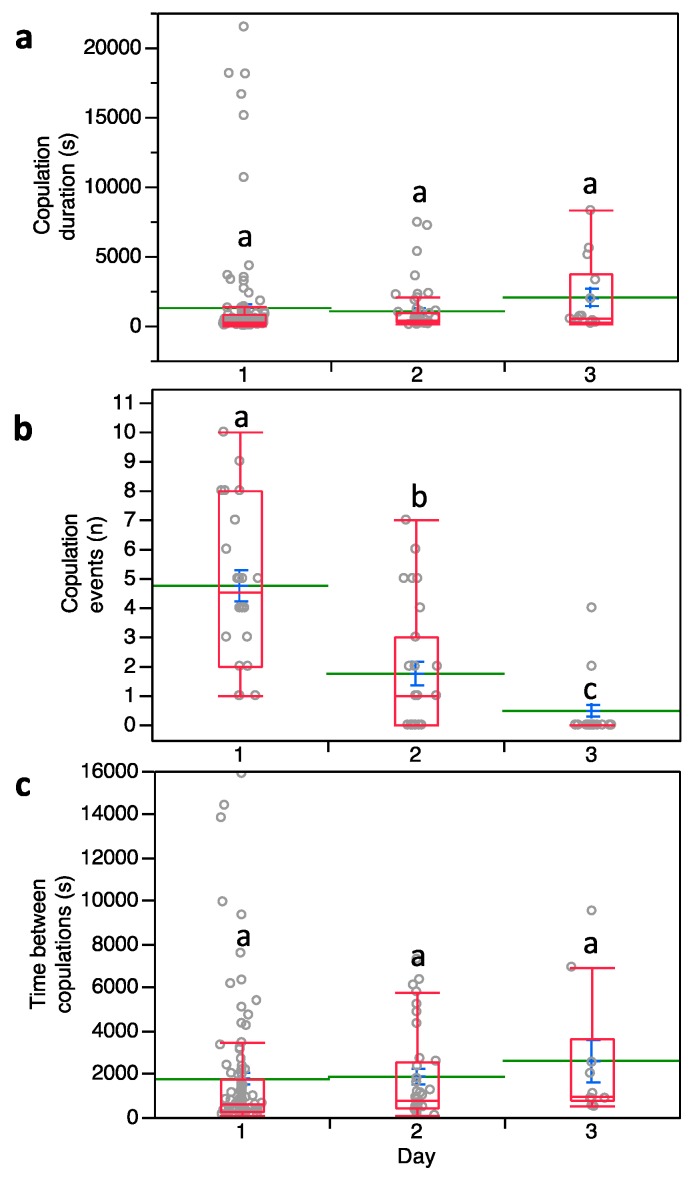
(**a**) Copulation duration, (**b**) number of copulation events and (**c**) time between copulations in *Pseudococcus calceolariae*. Box plots indicate the median (line) within each box and the range of dispersion (lower and upper quartiles and outliers). Green lines and blue T-bars indicate means and standard errors, respectively. Above each box plot, different letters indicate significant differences (general linear mixed model (GLMM), Tukey’s HSD test, *p* < 0.05).

Males with multiple mating events showed no significant differences in copula duration over three days of observations (*F*_2,172_ = 2.466; *p* = 0.088) (Figure 1a) while the copulation number per day was significantly affected (*F*_2,58_ = 39.549; *p* < 0.0001). The mean number of male successful copulation events per day was 4.73 the first day and then dropped to 1.73 and 0.47 on the second and third days, respectively (Figure 1b). Note that data from the fourth day of observations were not included in our analysis since only one male survived. The time between copulation events was not significantly affected by three subsequent days of repeated exposure to females (*F*_2,58_ = 0.839; *p* = 0.434) (Figure 1c).

**Figure 2:**
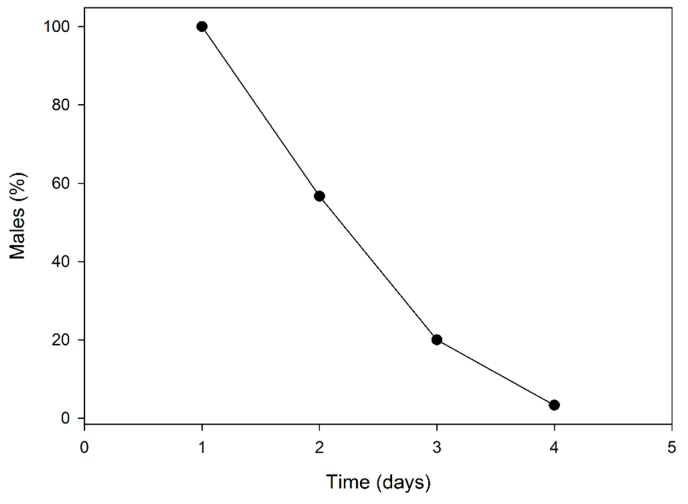
Longevity of *P. calceolariae* males subjected to multiple mating under laboratory conditions.

After the first day of mating, 56.7% of males survived and were then able to mate on the second day; 20% survived the second day and then mated during the third day. Just one male survived the third day and mated on the fourth day (Figure 2).

Overall, the maximum number of lifetime mating events was 13 and the daily number of mating events for each male ranged from 1 to 10, with a mean mating success of 92.8%. The mean number of copulation attempts during the whole life of *P. calceolariae* males was 7.0 ± 0.8 (mean ± SE), leading to 6.5 ± 0.7 fertilized females (n = 30).

### 3.2. Flight Tunnel Experiments

**Figure 3:**
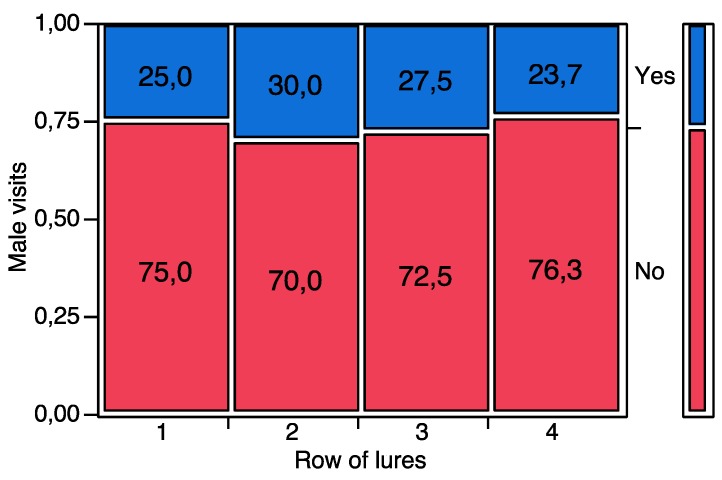
Contingency analysis between the rows of pheromone-baited lures (1 µg per lure, 16 lures, four per row across the wind) tested in the flight tunnel and the visits received by *P. calceolariae* males. The bar on the right represents the relative abundance (%) of male visits to a given row of lures over the total number of tested individuals. The number within each box shows the percentage of males visiting a given row of pheromone-baited lures. Yes = the male visited a given row of lures. No = the male did not visit a given row of lures. No significant differences were found among male visits to the different row of lures (*p* > 0.05).

When exposed to an array of rubber septa baited with synthetic pheromone, *P. calceolariae* males were attracted by upwind lures. In trial 1, with all 16 lures loaded with the same quantity of pheromone (1 μg), males did not show preferences for a specific rubber septum, visiting different rows of septa in a random way (*χ^2^* = 0.9422, *d.f. =* 3, *p* = 0.815) (Figure 3).

**Figure 4:**
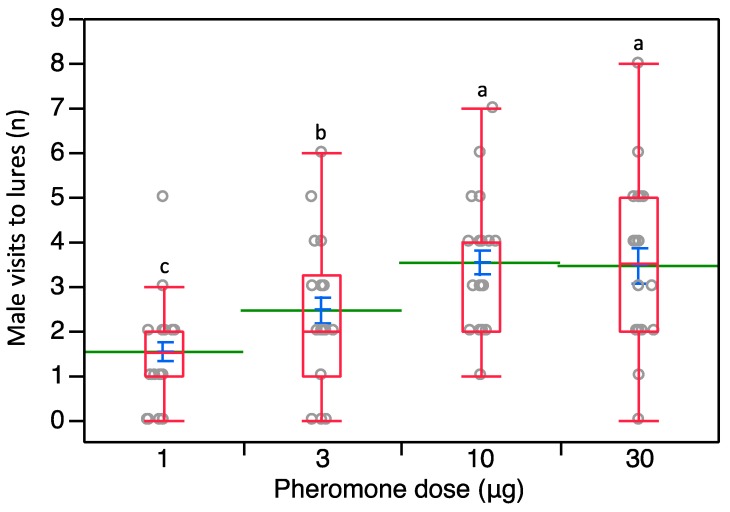
Distribution of *P. calceolariae* male visits to 16 lures organized in four rows with increasing loadings of sex pheromone going upwind (1, 3, 10, and 30 μg). Box plots indicate the median (line) within each box and the range of dispersion (lower and upper quartiles and outliers). Green lines and blue T-bars indicate means and standard errors, respectively. Above each box plot, different letters indicate significant differences (Steel-Dwass test, *p* < 0.05).

In trial 2, where we used an array with four different sex pheromone loadings, a significant effect of the tested loading was present (*χ*^2^ = 26.805, *d.f.* = 3, *p* < 0.0001). Most of the males showed positive chemotaxis towards the septa loaded with the highest quantities of pheromone, 10 and 30 μg (Figure 4). The highest number of visits per lure was achieved when testing 10 μg- and 30 μg-loaded septa (Figure 4); these septa showed significant differences over 3 μg- (Z = 2.677, *p* = 0.007; Z = 1.882, *p* = 0.05, respectively) and 1 μg-loaded lures (Z = 3.766, *p* = 0.001; Z = 4.790, *p* < 0.0001, respectively). Also, the number of male visits to 3 μg-loaded lures was significantly higher over 1 μg-loaded ones (Z = 2.426, *p* = 0.015) (Figure 4).

**Figure 5:**
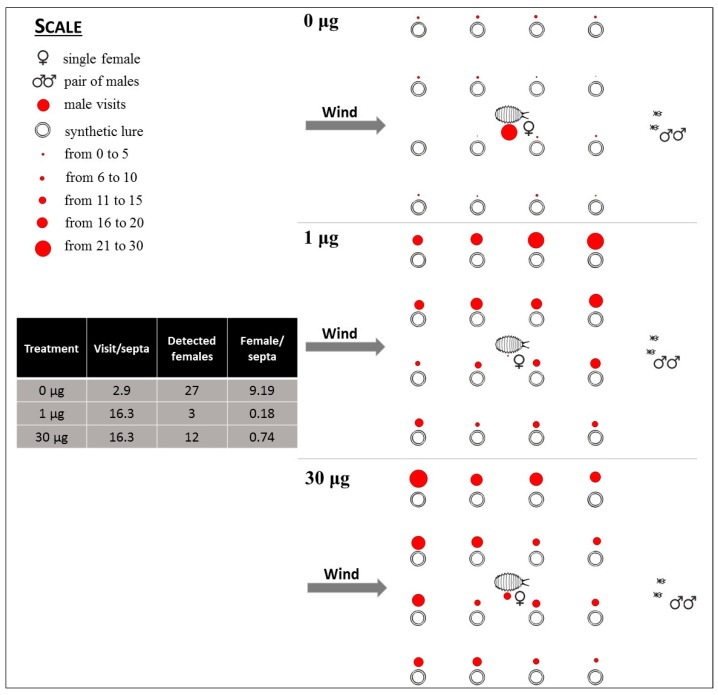
Distribution of visits of *P. calceolariae* males to the central female and to the 16 surrounding lures loaded with 0, 1, and 30 μg of synthetic sex pheromone. The diameter of spots represents the number of visits to each lure during 30 replicates, with two males released in each.

In trial 3, MD was mimicked at the flight tunnel scale by baiting all lures with 1 or 30 μg of *P. calceolariae* pheromone compared with unloaded controls. At 1 μg loading, the males successfully detected a female in the middle of the grid in just three cases out of 60 (5%) (Figure 5). No males attempted courtship or copulation. By increasing the loading of each bait to 30 μg, 12 out of 60 males detected the female (20%); none tried to fertilize her. In absence of the pheromone, 27 out of 60 males (45%) successfully detected the female and then mated and fertilized her.

**Figure 6:**
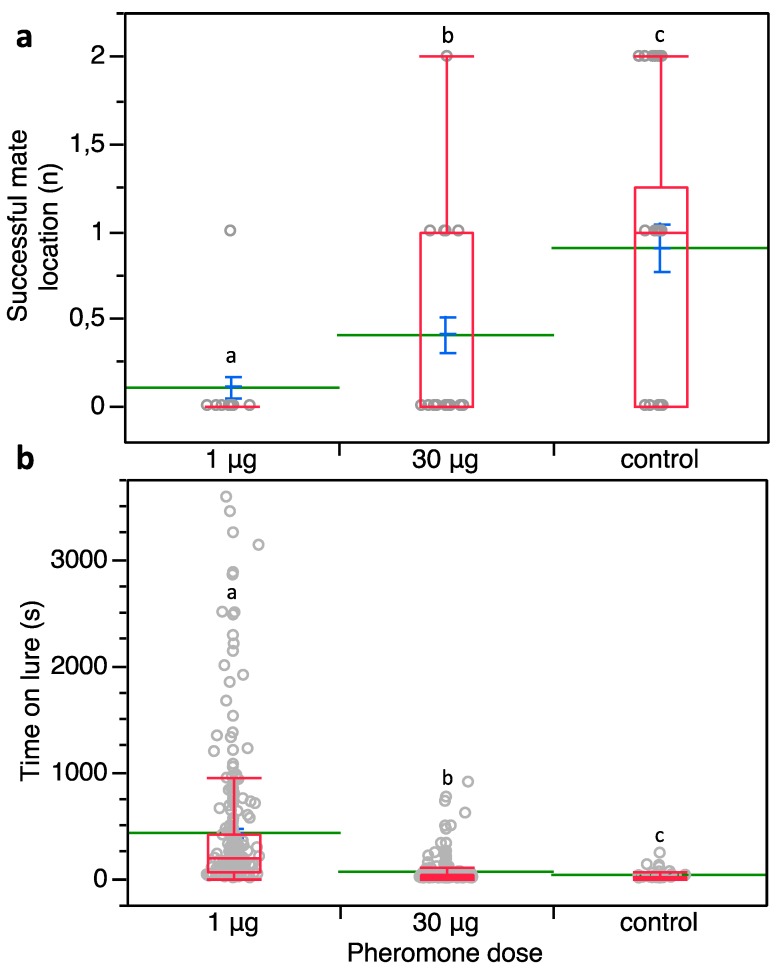
(**a**) Number of successful mate locations by *P. calceolariae* males surrounded by two different loadings of sex pheromone on 16 rubber septa at 2 cm spacing. (**b**) Time spent by *P. calceolariae* males on lures loaded with 1 or 30 μg of synthetic sex pheromone and control ones. Box plots indicate the median (line) within each box and the range of dispersion (lower and upper quartiles and outliers). Green lines and blue T-bars indicate means and standard errors, respectively. Above each box plot, different letters indicate significant differences (Steel-Dwass test, *p* < 0.05).

A significant effect of the sex pheromone loadings on successful mate location was detected (*χ*^2^ = 22.317, *d.f.* = 2, *p* < 0.0001) (Figure 6a). Successful mate detections were significantly reduced at both 1 and 30 μg per lure over the control (means: 0.10 and 0.40 versus 0.90 events; Z = 4.539, *p* < 0.0001 and Z = 2.647, *p* = 0.008, respectively). Successful mate detection events at 30 μg per lure were higher compared to those achieved at 1 μg loading per lure (Z = 2.436, *p* = 0.015) (Figure 6a).

Furthermore, the time spent by *P. calceolariae* males on the sex pheromone lures was significantly affected by the tested pheromone loading (*χ*^2^ = 235.964; *d.f.* = 2; *p* < 0.0001) (Figure 6b).

Indeed, the time spent on the lure was significantly higher at 1 μg of pheromone (421.38 ± 41.29 s, mean ± SE) when compared to the control (28.17 ± 5.70 s) (Z = −9.599; *d.f.* = 1; *p* < 0.0001) as well as to when 30 μg was used (56.39 ± 7.05 s) (Z = −14.202, *p* < 0.0001). Also, the time spent on the lure was significantly higher at 30 μg of sex pheromone over the control (Z = −2.813, *p* = 0.0049) (Figure 6b). In the control, the mean duration of copulation attempts was 1663.33 ± 262.73 s, while males did not attempt copulation when exposed to either sex pheromone loading.

## 4. Discussion

In mealybugs, the number of nymphs in a population depends on the number of mated females, which in turn depends on the number of males able to fertilize them. Multiple mating is common in *P. longispinus*, *P. ficus*, *P. viburni* [35], and *P. calceolariae* [32]. In the present research, we investigated the reproductive potential of *P. calceolariae* males in the context of simulated reduced-scale mating disruption.

Our research provides new insights into the number of copulations occurring during the male lifetime, their duration, and the time elapsing between copulations. The life expectancy and consequently the potential mating capability of *P. calceolariae* males was lower than that reported by Silva et al. [32]. The different results are probably due to various factors, i.e., number of males tested (100 against 30), RH conditions (50–70% in Silva et al. against 40% in our trial), and male life span (5–6 days in Silva et al. against 3–4 days in our case). In the days following the first mating, the number of copulation events strongly decreased. Several authors [29,35] have suggested that mated males need less time to successfully conclude forthcoming matings, putatively because they have gained experience after the first copulation event. Males may acquire more experience about how to approach a female, but, since they do not feed, they could potentially have less energy to devote to mating approaches [44]. By contrast, we did not find a significant difference in the daily mating duration nor in the time between copulations.

The multiple mating capacity of this mealybug potentially represents a limit to pheromone-based control techniques. Indeed, in mass trapping and lure and kill techniques, even if we eliminate a high number of males, the remaining ones would be able to fertilize several females, allowing the perpetuation and increase of the population [26]. Studies carried out in citrus orchards infested by *P. citri* have demonstrated that mass-trapping using pheromone-baited traps can produce a significant reduction of males, but this was not enough to decrease infestation on the plants [45]. Considering other examples on extremely different insect species, such as *Calliphora vicina* Robineau-Desvoidy (Diptera, Calliphoridae), mass trapping has proved to be an effective technique, probably because the traps were baited with an attractant that allowed for the catching of males (20%) but especially many females (80%) [46]. Moreover, the costs related to attractive products and the labour required for the installation of numerous traps can be very high [47]. Another potential control system of this pest could be MD. Indeed, the latter has been found effective for the management of the vine mealybug, *P. ficus* [16,20,21,22,36,37]. Earlier tests revealed that habituation to the sex pheromone did not occur in *P. calceolariae* [31]. Our flight tunnel data pointed to the potential use of MD against this mealybug species by analyzing the male searching behavior towards females in the presence of multiple sources of synthetic sex pheromone.

The flight tunnel has been used to study several facets of insect behavior and in chemical ecology [48,49,50,51]. The flight tunnel can also be used to assess MD in Lepidoptera [52,53]. In this framework, our study used a flight tunnel to investigate MD in Hemiptera. We reproduced, on a small scale, conditions that are essentially comparable with mating disruption in the field, where the male emerges at a close range to the females in the colony. Results were promising from the perspective of reducing mating in the presence of an overabundance of *P. calceolariae* sex pheromone. In fact, as demonstrated by Lentini et al. [54] on *P. ficus*, even a mating delay has positive effects if mating is delayed >7 days. Field trapping trials by Unelius et al. [55] have revealed that synthetic lures are much more attractive than calling females to *P. calceolariae* males. Our results from flight tunnel trial 2 support this observation, showing that searching males prefer to arrest on the lure loaded with the highest loading of pheromone.

The MD tests using synthetic sex pheromone have shown success in obscuring the presence of the female compared to the control, in which the female was detected and fertilized by males. Testing both sex pheromone loadings, no females were approached or fertilized, while in the control group males performed prolonged copulation attempts and all the females were fertilized. Under MD conditions, a few males found the females and this could raise some doubts about performance in the field, since spacings were very close (2 cm). However, after finding the female, the males changed direction, and thus the observed events may be due to male random movements. In the trial testing the effect of 30 μg of synthetic sex pheromone, more males detected the female, showing a significant difference with male performances in trials testing 1 μg as well as the negative control. Probably the greater disruption, generated by a high pheromone quantity in the air, caused this difference by stimulating the males to walk much more instead of dwelling on the lures. Indeed, they spent an average of 56 s on the lure. The above-cited increased male activity could boost the chance of finding a female. On the contrary, under MD with 1 μg, males spent about 420 s per lure, eight times more if compared to time spent in trials testing 30 μg. Furthermore, comparing the male copulation attempts, it was evident that in the control males spent a lot of time trying to approach females, while in presence of both pheromone loadings, males did not perform copulation attempts, spending all the time above lures or walking. This confirms the observations carried out by Silva et al. [29] on the mating behavior of this species. After finding the female during the courtship phase, the male explores the female body by drumming it with the antennae; in response, the consenting female raises her abdomen to accept mating. Starting from this assumption, we can suppose that the loadings were not subjected to copulation attempts because they were not recognized as females. Comparable results have been achieved on *Grapholita molesta* Busck (Lepidoptera, Tortricidae) males in presence of increasing loadings of the sex pheromone [56]. Further research is still needed to investigate the behavior of *P. calceolariae* in response to the increasing amount of pheromone to identify the most appropriate loading to be used for MD purposes. At the same time, it would be useful to increase knowledge on the use of potential biological control agents of this species [57] as already done for other mealybugs [58].

Our results on *P. calceolariae*, as well as the evidence from field tests outlining the effectiveness of MD on *P. ficus* in California, Italy, Israel, and Tunisia [20,21,22,36,37,59], constitute a basis for undertaking further investigations into the potential of MD or male removal for *P. calceolariae* management. While MD applied to another mealybug species has led to good results at a large scale, other techniques such as mass trapping and lure and kill have similar limitations [45,60,61] and remain to be investigated. The flight tunnel can represent a fundamental tool to assess the MD effectiveness at a small scale in a wider range of species beyond Lepidoptera, where it has been noted that very few mechanistic studies are usually undertaken [31]. This would provide rapid and relatively cheap preliminary results of MD efficacy on a given pest. Certainly, the field conditions are more complex than those in the flight tunnel, since a greater number of variables are involved in field trials, including immigration from upwind by crawlers, requiring very large plots and multiple measures of population size to demonstrate efficacy. Nevertheless, flight tunnel experiments can provide important information of the possible efficacy of MD, as well as assess the absence of habituation and identify the risk of overstimulating male searching from high pheromone loadings. Indeed, this may be a research topic to be considered in further MD research, involving evaluating whether MD formulations releasing plumes of pheromones, such as aerosol or sprayable technologies, might be more effective over hand-held dispensers releasing higher pheromone loadings per surface area unit. However, at present there does not seem to be any possible way to make the *P. calceolariae* pheromone in large amounts at a cost that would be competitive with insecticides. This is a major and potentially insurmountable problem that could easily prevent MD ever being used on a large scale for this insect and future research into field applications of the sex pheromone of this species should focus on male removal strategies [26] and monitoring.
